# Preparation of Modified Fluorographene Oxide with Interlayer Supporting Structure

**DOI:** 10.3390/polym13183126

**Published:** 2021-09-16

**Authors:** Chengbing Yu, Kaiqin Shi, Jinyan Ning, Jun Liu

**Affiliations:** 1 School of Materials Science and Engineering, Shanghai University, Shanghai 201800, China; skqin112@shu.edu.cn; 2 Materials Genome Institute, Shanghai University, Shanghai 200444, China; jyning@t.shu.edu.cn; 3 Shanghai Institute of Quality Inspection and Technical Research, Shanghai 201114, China

**Keywords:** fluorographene oxide, p-phenylenediamine, amine groups, interlayer supporting structure, reactive modifier

## Abstract

Fluorinated graphene (FGi) is easy to agglomerate, after which it turns into a curly and wavy shape, which results in a great decrease in the properties of the resultant composite materials and coatings. In this study, fluorinated graphene oxide (FGO) modified with p-phenylenediamine (PPD) was prepared, but with a view to avoid its agglomeration and retain a sheet-like structure. Through the reaction between PPD and the epoxy groups of FGO, the modified FGO with an amino group (N-PGO) had a larger interlayer d-spacing than FGO. The stability of N-PGO was also improved, and nitrogen, fluorine, oxygen, and carbon were evenly distributed in the N-PGO sheets. All the results indicate that PPD can act as an effective spacer to separate graphene sheets for good anti-agglomeration properties. This method produced modified graphene with fluorine, amino, and carbonyl groups. It shows potential in introducing N-PGO as a reactive modifier in composite materials and coatings for a variety of industrial applications including waterborne epoxy materials.

## 1. Introduction

As seen in the first two-dimensional atomic crystal available to us [1], graphene is made out of carbon atoms arranged in a hexagonal honeycomb structure [2,3]. Graphene derivatives mainly include graphene oxide (GO), fluorinated graphene (FGi), and hydrographene, which have strong characteristics that other carbon-based materials do not have, such as a high specific surface area, high electron mobility, and a high barrier. Each graphene derivative with special chemical and structural characteristics exhibits different properties and uses; in particular, graphene derivatives with active groups (such as amino, epoxy, and hydroxyl) allow a wide variety of chemical modification or functionalization routes with other materials [4]. Therefore, graphene derivatives are appealing materials in many fields, such as electronics, composite materials, clean energy devices, biology, and medicine, due to the tunability of both their chemical composition and sheet size [5].

Active research on FGi has revealed various excellent properties such as super-hydrophobicity, high thermal and chemical stability, high Young’s modulus, and high mechanical strength [6,7]. Such fascinating properties have triggered a broad interest. For example, FGi has the potential to be applied for novel nano-electronic devices due to its high carrier mobility [8,9], while for biomedical application, fluorinated graphene oxide (FGO) shows its significant effect on the nuclear elongation of mesenchymal stem cells [10]. FGi-based coatings show excellent weather resistance [11] and enhanced lubrication performance [12,13]. The application of FGi also involves fields such as supercapacitors, electrochemistry, and amphiphilic membrane surfaces [7,14,15,16].

However, the study of FGi has been mainly focused on synthesis and applications, and there is no research on improving its anti-agglomeration properties. Compared with conventional graphene, FGi agglomerates more easily and becomes a curly and wavy shape due to strong van der Waals forces [17], which makes it difficult to disperse uniformly and improve the properties of the matrix greatly. Therefore, its performance in composite materials and coatings cannot be fully utilized, and it acts only as a conventional filler. It is thus necessary to keep the FGi in a sheet-like structure and prevent it from agglomerating.

There are two main methods to prevent the agglomeration of graphene sheets, which differ depending on how the separator is introduced. The first method utilizes physical adsorption to introduce nanoparticles such as gold [18], silicon [19], or platinum [20] between the sheets and separates the sheets by a physical barrier. The second method involves a chemical reaction between the functional groups in the surface of the graphene sheets and the separator [21,22,23]: there are already some reports on using different separators to obtain useful graphene derivatives. Vermisoglou [24] reported well-defined graphene derivatives differing in chemical composition but with similar morphologies by controlling the reaction time of 5-aminoisophthalic acid and FGi. Zaoralová [25] synthesized highly N-doped graphene by the reaction of FGi with NaNH_2_ under mild and sustainable conditions. p-Phenylenediamine (PPD) can also act as an effective spacer to separate graphene oxide (GO) sheets. Sk [26] used PPD to detach the graphene oxide layers with two -NH_2_ groups, in order for two or more GO layers to form a layer-by-layer assembly structure through the reaction between the amine groups and the epoxy groups, and for the interlayer structure to act as a spacer to prevent GO sheets’ agglomeration. Lu [27] also modified GO sheets with PPD through the epoxy ring-opening reaction and observed a loose and cross-linked microstructure. Compared with GO, FGO is easier to agglomerate and turn into a curly and wavy shape, but no research has discovered a method to introduce the separator in FGO. 

The improved Hummers method is an effective method widely used for the synthesis of GO [28,29] and FGO [30,31], which can introduce oxygen-containing groups to the surface of sheets while introducing thermally exfoliated graphite into graphene. In this work, various oxygen-containing groups obtained in FGO were used for the first time, and after the thermal exfoliation process, FGO was modified with PPD to construct an interlayer support frame to prevent agglomeration, which is a simple and feasible route. The present study reveals a method for modifying FGO with PPD to obtain N-PGO with anti-agglomeration properties.

## 2. Materials and Methods

### 2.1. Materials and Chemical Reagents

Fluorinated graphite (fluorine content 40%) was supplied by CarFluor Chemicals Co., Ltd. (Shanghai, China). Sodium nitrate (NaNO_3_, ≥99%), sulfuric acid (H_2_SO_4_, 98%), potassium permanganate (KMnO_4_, ≥99.5%), hydrogen peroxide (H_2_O_2_, ≥30%), hydrochloric acid (HCl, 37%), p-phenylenediamine (PPD, ≥99%), and ethanol (≥95%) were purchased from Guoyao Group Chemical Reagent Co., Ltd. (Shanghai, China). Deionized water was processed in a Milli-Q water purification system.

### 2.2. Preparation Methods

#### 2.2.1. Preparation of FGO

**Scheme 1:**
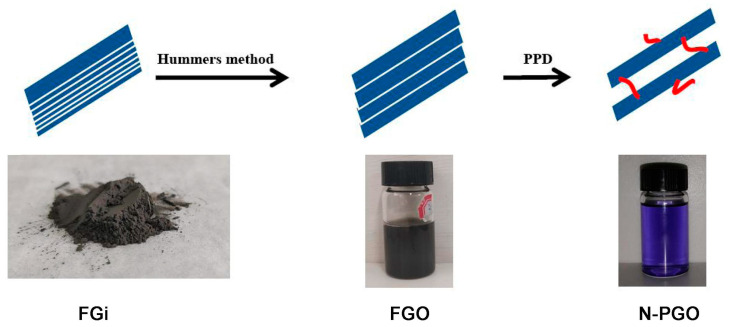
Preparation process of N-PGO.

An improved Hummer’s method [32,33] was used to prepare FGO by adding FGi (3 g) and NaNO_3_ (1.5 g) to H_2_SO_4_ (70 mL) in an ice bath. Then, they were dispersed by magnetic stirring in order for FGi to be completely soaked and pre-oxidized by H_2_SO_4_. After about 1.5 h, KMnO_4_ (12 g) was slowly added to the mixture under continuous stirring, while the temperature was controlled below 10 °C. The mixture was stirred in an ice bath for 2 h and for another hour in a water bath at 35 °C. Following this step, deionized water (140 mL) was added dropwise to the mixture, and the temperature reached 90–98 °C due to the exothermic reaction. Deionized water (500 mL) and H_2_O_2_ (20 mL) were then added after the mixture cooled to room temperature. The mixture including the floatings and dispersion in the solution was first washed with HCl (5 wt%) and then with deionized water, followed by centrifugation at 10,000 rpm to remove excess acid and inorganic salts. The final FGO was freeze dried for 3 days (Scheme 1).

#### 2.2.2. Preparation of N-PGO

PPD (1 g) was added into deionized water (100 mL) and stirred until it was completely dissolved to prepare a diamine solution. FGO (0.5 g) was then added into deionized water (250 mL) and ultrasonicated for 2 h to obtain a homogeneous dispersion. The diamine solution was slowly added over 1 h to the FGO dispersion in a water bath at 60 °C, and the mixture was stirred for another 6 h until the solution turned dark purple. Then, the mixture was cooled to room temperature and washed with deionized water and ethanol by centrifugation at 10,000 rpm to remove excess PPD. The final N-PGO was freeze dried for 3 days (Scheme 1).

### 2.3. Characterization and Corrosion Analysis

The functional groups of N-PGO were analyzed by a Nicolet 380 Fourier transform infrared spectroscopy (FTIR) (Thermo-Fisher Scientific, Waltham, MA, USA) and an ESCALAB 250Xi X-ray photoelectron spectroscopy (XPS) (Thermo-Fisher scientific, Waltham, MA, USA). The interlayer spacing was investigated by X-ray diffraction (XRD) (D/MAX 2500V, Rigaku Industrial Corporation, Takatsuki-shi, Osaka, Japan). The particle size was obtained by a nanosizer (Nano ZS90, Malvern Instruments, Malvern, UK). The morphologies of N-PGO were characterized by high-resolution transmission electron microscopy (HR-TEM), selected area electron diffraction (SAED), and energy-dispersive spectroscopy (EDS) on a JEOL 200CX transmission electron microscope (Akishima-shi, Tokyo, Japan).

## 3. Results and Discussion

FGO obtained by an improved Hummers method had various oxygen-containing groups that allowed covalent functionalization. The interlayer support structure was constructed through the reaction between the amine groups of PPD and the epoxy groups on the surface of the FGO sheets (Scheme 1). It increased the distance between the layers to avoid agglomeration and prevent the sheets from becoming a curly and wavy shape. 

**Figure 1:**
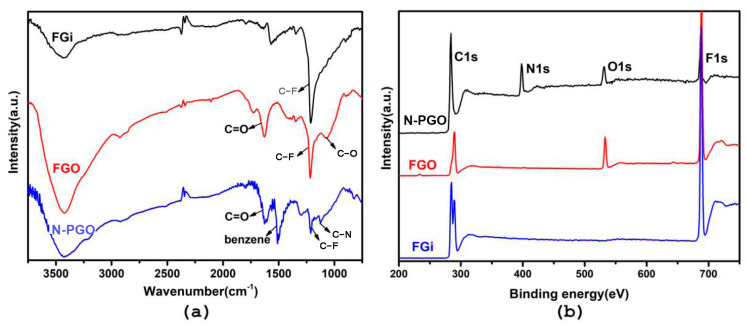
FTIR (**a**) and wide-scan XPS (**b**) spectra of FGi, FGO, and N-PGO.

The FTIR spectra of FGi, FGO, and N-PGO were measured to study the changes in the functional groups in each sample (Figure 1a). The three spectra feature the same characteristic peaks at 1220 cm^−1^ and 3417 cm^−1^, corresponding to the stretching vibrations of C–F and –OH [2], respectively, which reveals that the fluorine atoms in graphite fluoride remained after oxidation and subsequent modification. The prepared N-PGO also retained its special properties due to its C–F bonds. The FGO spectrum exhibits two new peaks at 874 cm^−1^ and 1730 cm^−1^, which are assigned to epoxy groups and –COOH, respectively. During liquid-phase modification, –COOH transformed to –CONH– by the chemical reaction between –NH_2_ and –COOH. The SN2 reaction of the -NH_2_ group to the epoxy ring resulted in its opening, with subsequent formation of the –CH(OH)–CH_2_–(NH)– moiety. The absorption peaks for C–O, C–N, and aromatic amines were found at 1305 cm^−1^, 1123 cm^−1^, and 1305 cm^−1^ (C–N stretching) [34], respectively. The sharp absorption peak at 1511 cm^−1^ was identified as benzene ring vibrations due to the introduction of PPD [35]. As shown above, the FTIR spectra of FGi, FGO, and N-PGO indicate that PPD was grafted onto the layer surfaces via a ring-opening reaction.

To determine the surface chemical compositions, wide-scan XPS spectra were obtained (Figure 1b). The characteristic peaks at 284 eV and 689 eV correspond to C1s and F1s, respectively. The characteristic peak at 533 eV corresponds to O1s, and the characteristic peak at 398 eV corresponds to N1s. This indicates that oxygen was introduced into FGi through the improved Hummers method, and nitrogen was then introduced into N-PGO through the modification reaction. Fluorine was retained in N-PGO from FGi, suggesting its unique performance was also retained. The element composition can directly reflect the differences in various materials. As shown in Table 1, after the modification reaction, the oxygen content of N-PGO decreased from 8.67 to 4.68%, which can be attributed to the transformation of –COOH and carbonyl groups (>C=O) to –CO–NH–C_6_H_4_–NH– groups and phenylenediamine imine structures >C=N–Ar. The nitrogen content increased from 0 to 8.81%, and the carbon content also increased. This indicates that nitrogen was introduced, but the benzene ring still worked as a framework to build layer-supporting structures between the graphene sheets.

**Figure 2:**
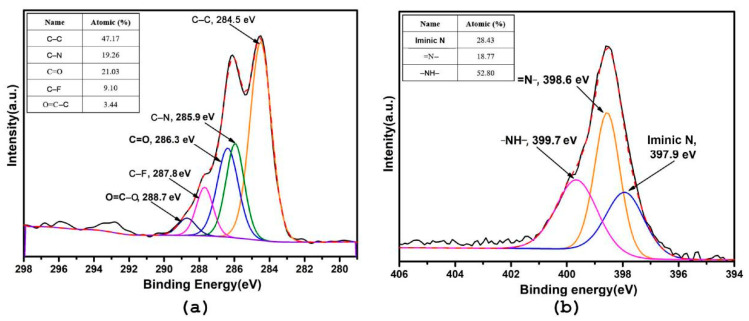
C1s (**a**) and N1s (**b**) signatures of N-PGO by XPS.

Furthermore, the XPS peak differentiation imitating C1s for N-PGO (Figure 2a) showed a component at 285.9 eV, which can be attributed to C–N. In addition, in the XPS peak differentiation imitating N1s for N-PGO (Figure 2b), the component of iminic N appears with binding energies located at 397.9 eV (iminic N refers to N atoms at the edge of graphene sheets with bonds to two carbon atoms [36]). The analysis indicates that the aforementioned ring-opening reaction occurred during the functionalization process, which is consistent with the FTIR results. 

**Figure 3:**
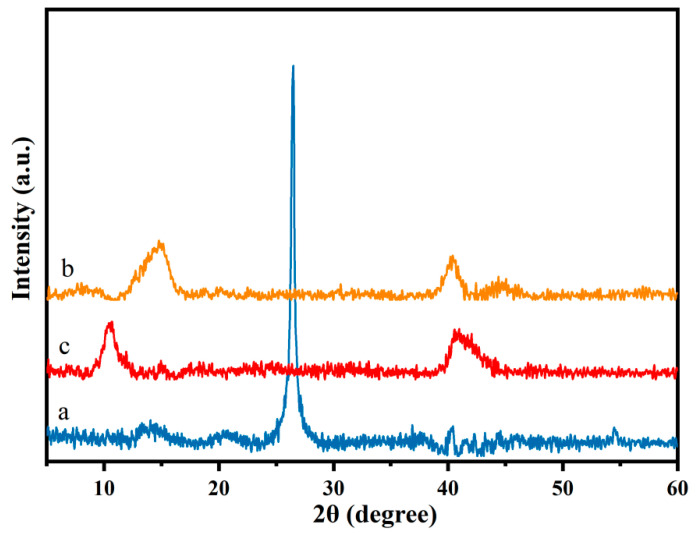
XRD spectra of FGi (**a**), FGO (**b**), and N-PGO (**c**).

The interlayer d-spacing could be calculated through the Bragg equation as the XRD patterns of FGi, FGO, and N-PGO (Figure 3) illustrated a shift in the peak position. FGi showed a peak at approximately 26.5°, corresponding to a d-spacing of 3.36 Å. After exfoliation, the peak shifted to approximately 14.9°, corresponding to a d-spacing of 5.94 Å, which indicates that the treatment with the improved Hummers method greatly increased the distance between the layers. For N-PGO, the peak was around 10.5°, and the d-spacing was 8.42 Å. The increase in the d-spacing implies the grafted PPD acted as a spacer to separate the graphene sheets. 

The particle size of FGO and N-PGO was tested after standing for 24 h (Table 2). The particle size of the graphene sheets decreased from 438.9 to 293.3 nm after modification. A plausible explanation is that the PPD is attached to the surface of FGO via the reaction between the amino group and epoxy during the process; the resulting interlayer support frame slows down the agglomeration of the graphene sheet and allows it to retain a sheet-like structure. The zeta potential also increased significantly from 15.3 to 38.8 mV, evidencing that the stability of N-PGO was improved.

**Figure 4:**
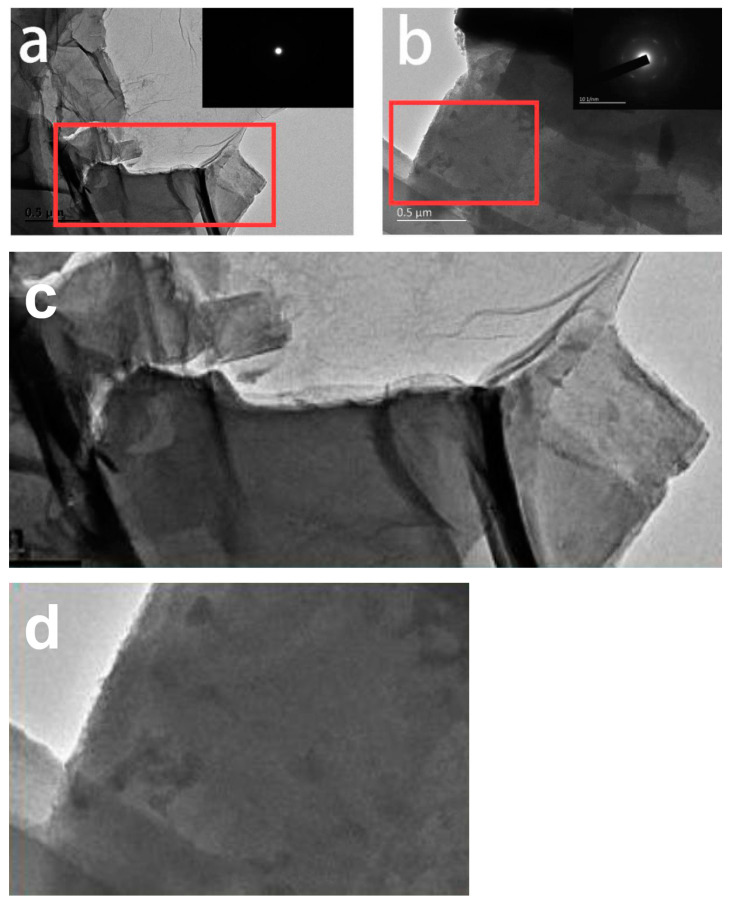
TEM images of FGO (**a**), N-PGO (**b**), partially enlarged image (**c**) and (**d**) of FGO and N-PGO, respectively.

TEM was used to observe the microstructures of the samples (Figure 4). The SAED images of FGO and N-PGO show regular hexagonal diffraction, proving that both have a regular graphene structure. The FGO sheet has a transparent thin-layer structure, and the edges of the folds on the surfaces are curled and folded, along with some overlap between the sheets. After modification, the TEM image of the N-PGO sheets shows a stretched sheet structure, and the surfaces and edges of the sheets are smooth, while the boundaries between the layers are visible. Previous research on the modification of graphene and its derivatives by PPD demonstrated that the interlayer supporting structure formed between the sheets could expand the layer spacing and cause the sheets to form a stacked layered structure [37]. In this experiment, the interlaminar forces of N-PGO were weakened due to expansion of the interlayer distance, meaning the sheets existed in a stretched form.

**Figure 5:**
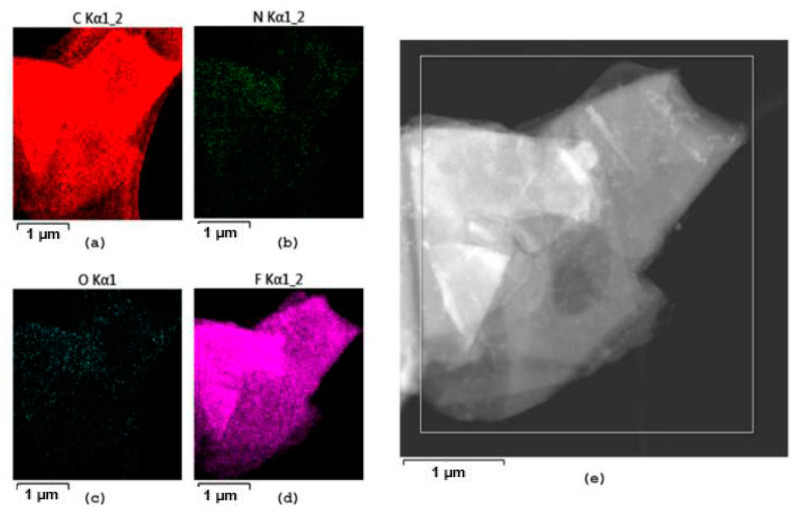
In situ EDS mapping patterns of N-PGO for C (**a**), N (**b**), O (**c**), and F (**d**); STEM-HAADF image of N-PGO (**e**).

Elemental mapping by EDS (Figure 5) was used to determine the elemental distribution of each sample. Figure 5a–d present the distributions of C, N, O, and F on N-PGO samples, respectively. No missing blocks are observed in the image where the bright spots of each element are displayed. Figure 5e shows that the surface of N-PGO was flat and smooth, with multiple disordered stacked layers. The scanned image of the selected area shows that the elements were distributed on the N-PGO sheets. The existence of nitrogen and oxygen suggests that the oxidation and modification reactions occurred successfully. The shape of the elemental maps of N-PGO is similar to the scanning area in the TEM images. The sheets were stacked while maintaining a flat surface, revealing that the supporting structure formed between the layers maintained the stretching of the sheets.

The present study reported a simple two-stage route to prepare N-PGO with anti-agglomeration properties via PPD modification: more specifically, FGO was first prepared from FGi by an improved Hummers method and then modified with PPD through a liquid-phase reaction to obtain N-PGO sheets (Scheme 1). Compared to previous reports, our product has fluorine, amino, and carbonyl groups and is soluble in water. The C-F bond in the graphene sheet broadens its applications in various fields, and the amino and carbonyl groups can enhance its compatibility with the matrix. In particular, the separator between the layers not only creates a cross-linked structure with more interlayer space to avoid agglomeration but also keeps the sheets smooth and unfolded, meaning they can fully exert their great advantage in composite materials and coatings.

## 4. Conclusions

In summary, this study proposed a facile method to modify FGO and the resultant N-PGO with anti-agglomeration property benefits from the interlayer support structure formed by the reaction between PPD and the epoxy groups of FGO. The N-PGO sheets had a smooth surface and a larger interlayer space. Moreover, the oxygen-containing groups and nitrogen-containing groups of N-PGO, which have active chemical reactivity, enhanced its compatibility with the composite materials and coatings. Specifically, the presence of the primary amino groups of N-PGO makes it very suitable for waterborne epoxy materials.

## Figures and Tables

**Table 1:** Element compositions of FGi, FGO, and N-PGO.

Samples	Element Compositions (%)			
	C1s	O1s	N1s	F1s
FGi	64.42	0	0	35.58
FGO	61.49	8.67	0	29.84
N-PGO	69.37	4.68	8.81	17.14

**Table 2:** Particle size and zeta potential of FGi, FGO, and N-PGO in water.

Samples	Z-Average (r. nm)	Zeta Potential (mV)
FGi	-	-
FGO	438.9	−15.3
N-PGO	293.3	−38.8

## Data Availability

The data that support the findings of this study are available from the corresponding author (C. Yu, J. Liu) upon reasonable request.
